# Sonication of retrieved implants improves sensitivity in the diagnosis of periprosthetic joint infection

**DOI:** 10.1186/s12891-019-3006-1

**Published:** 2019-12-26

**Authors:** Petri Bellova, Veronika Knop-Hammad, Matthias Königshausen, Eileen Mempel, Sven Frieler, Jan Gessmann, Thomas A. Schildhauer, Hinnerk Baecker

**Affiliations:** 1 Department of Orthopedic and Trauma Surgery, Surgical Clinic, BG University Clinic Bergmannsheil Bochum, Bürkle-de-la-Camp Platz 1, 44789 Bochum, Germany; 2 Department of Microbiology, BG University Clinic Bergmannsheil Bochum, Bochum, Germany

**Keywords:** Periprosthetic joint infection, PJI, Hip, Knee, Sonication, Culture, Sensitivity, Specificity, Pathogen

## Abstract

**Background:**

Sonication is a valuable tool in the diagnosis of periprosthetic joint infections (PJI). However, conditions and definition criteria for PJI vary among studies. The aim of this study was to determine the diagnostic performance (i.e., specificity, sensitivity) of sonicate fluid culture (SFC) against periprosthetic tissue culture (PTC), when using European Bone and Joint Infection Society (EBJIS) criteria.

**Methods:**

From March 2017 to April 2018, 257 implants were submitted for sonication. PJI was defined according to the EBJIS criteria as well as according to the International Consensus Meeting criteria of 2018 (ICM 2018). Only cases with at least one corresponding tissue sample were included. Samples were cultured using traditional microbiological plating techniques. Sensitivity and specificity were determined using two-by-two contingency tables. McNemar’s test was used to compare proportions among paired samples. Subgroup analysis was performed dividing the cohort according to the site of PJI, previous antibiotic treatment, and time of manifestation. Prevalence of pathogens was determined for all patients as well as for specific subgroups.

**Results:**

Among the 257 cases, 145 and 112 were defined as PJI and aseptic failure, respectively. When using the EBJIS criteria, the sensitivity of SFC and PTC was 69.0 and 62.8%, respectively (*p* = .04). Meanwhile, the specificity was 90.2 and 92.9%, respectively (*p* = .65). When adopting ICM 2018 criteria, the sensitivity of SFC and PTC was 87.5 and 84.4% (*p* = .63) respectively, while the specificity was 85.1 and 92.5% (*p* = .05), respectively. The most commonly identified pathogens were coagulase-negative staphylococci (26% overall), while 31% of PJI were culture-negative and 9% polymicrobial.

**Conclusions:**

SFC exhibited significantly greater sensitivity versus PTC when using the EBJIS criteria. Nevertheless, the diagnosis of PJI remains a difficult challenge and different diagnostic tools are necessary to optimize the outcome.

## Background

Periprosthetic joint infections (PJI) pose a major challenge in orthopedic surgery and traumatology. Every infection is a complication associated with multiple surgeries, prolonged hospitalization and significantly increased morbidity, while placing a major burden on the economy [1].

Accurate diagnosis is crucial for the successful treatment of PJI. However, it may be difficult owing to the fact that there is no single test available with 100% sensitivity [2]. Thus, a combination of factors including clinical signs, laboratory results from peripheral blood and synovial fluid, microbiological culture and histological evaluation of periprosthetic tissue is necessary. The Musculoskeletal Infection Society (MSIS) and the Infectious Diseases Society have developed criteria to standardize the definition of PJI [3]. These criteria have been modified since their establishment [4, 5] leading to an increase in diagnostic confidence. Most recently, the ICM criteria were established as part of the 2018 International Consensus Meeting [5].

**Fig. 1:**
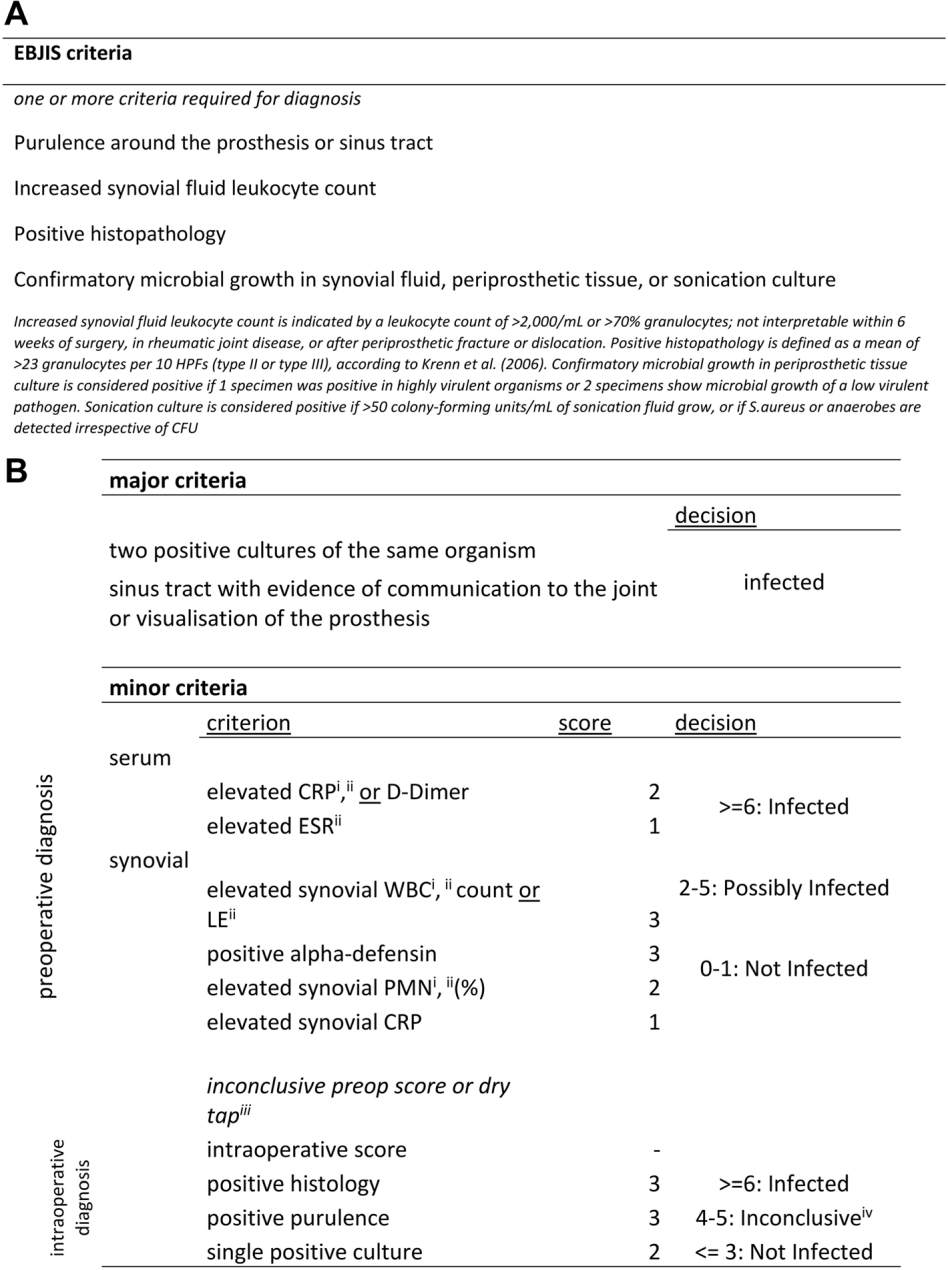
**a** EBJIS criteria. **b** 2018 ICM criteria. ^i^ **thresholds** .^ii^ CRP, C-reactive protein; ESR, erythrocyte sedimentation rate; WBC, white blood cell count; LE, leucocyte esterase; PMN, polymorphonuclear. ^iii^ For patients with inconclusive minor criteria, operative criteria can be used to fulfill definition for PJI. ^iiii^ Further molecular diagnostics such as next generation sequencing should be considered

Previous criteria were designed for the diagnosis of definitive PJI. However, there is a considerable number of low-grade infections presenting with only subtle clinical and nonconfirmatory microbiological findings. These low-grade infections are often missed by common criteria. Based on this notion, a modified classification system for the diagnosis of PJI was proposed by Swiss Orthopaedics and the Swiss Society of Infectious Diseases (SOSSID) [6, 7]. In 2017, the SOSSID criteria were proposed by the European Bone and Joint Infection Society (EBJIS). These criteria use lower cutoff values for synovial fluid count and include sonication (Fig. 1).

Although tissue culturing has been the gold standard for the detection of causative pathogens, this approach is characterized by a high rate of false negatives [8, 9]. The advantage of sonication lies in its capability to disrupt biofilms and thus, increase the number of microorganisms available for culture [10]. This allows for the administration of a more specific antibiotic treatment. Although several studies have shown improved sensitivity following the use of sonication, study conditions and definitions of PJI vary considerably.

The aim of this study was to determine the sensitivity and specificity of sonicate fluid culture (SFC) compared with periprosthetic tissue culture (PTC), using the EBJIS criteria as reference standard. In addition, for direct comparison, we defined PJI according to the current ICM criteria.

## Methods

We retrospectively reviewed data of retrieved joint prostheses from March 2017 to April 2018. During this period, 257 implants were submitted for sonication. Data were obtained from the electronic records of the hospital. The local Ethical Review Committee approved this study (19–6603).

Inclusion criteria were the availability of sonicate fluid culture (SFC) for any prosthesis or prosthesis component between March 2017 and April 2018. Exclusion criteria were lack of corresponding tissue culture samples (at least 1 necessary for inclusion) and retrieval of any hardware other than prostheses or prostheses components.

For the definition of infection according to the EBJIS and ICM criteria, data including patient demographics (age, gender), surgery performed (type and date), clinical information (presence of a sinus tract or pus), microbiological and histopathological findings were obtained (Fig. 1a, b). Furthermore, information regarding preoperative administration of antibiotics, manifestation of the infection and duration of symptoms was collected. According to the respective definition criteria, cases were divided into PJI or aseptic failure (AF).

### PTC

Intraoperative sampling of periprosthetic tissue was performed from the area macroscopically most suspicious of infection. Multiple samples were obtained in the vicinity of the implant. Cases lacking tissue samples were excluded from the analysis. In the microbiological laboratory, samples were prepared using forceps and scalpel under laminar air flow. Aliquots of the tissue were subsequently placed on different aerobic and anaerobic culture plates, and growth media (blood agar, chocolate agar, Schaedler agar, brain-heart infusion, Wilkens–Chalgren infusion). Culture was performed under human body temperature conditions (37 °C) for 14 days.

### SFC

Explants were placed in sterile polypropylene containers that were opened in the operating room immediately prior to component explantation. In the laboratory, explants were immersed in Ampuwa® (Fresenius Kabi Deutschland GmbH; Bad Homburg, Germany) solution and treated in an ultrasonic bath (BactoSonic; Bandelin, Berlin, Germany) for 60 s at 80% *P* = 160 W. Subsequently, 10 ml of sonicate fluid were placed in aerobic and anaerobic blood culture bottles, followed by culture.

### Synovial fluid culture of preoperative joint aspiration (PJA)

Synovial fluid was obtained under sterile conditions. The skin was disinfected thrice, and covered with sterile drapes. A skin incision was performed prior to aspiration. The retrieved joint fluid was subsequently divided into aliquots for microbiological and for cell counting analysis. White blood cell (WBC), polymorphonuclear neutrophil (PMN) and differential blood count were performed. The thresholds for WBC and PMN counts varied between the different criteria **(**Fig. 1**)**.

### Periprosthetic membrane (PM)

Histopathological examination of the PM was performed using the consensus classification established by Krenn and Morawietz [11]. A type 2 (septic failure) or type 3 (combined type).

PM was classified as infected.

As a retrospective study, not all diagnostic tools were deployed for each patient.

Subgroup analysis was performed dividing the cohort as follows:

1. Hip versus knee versus other
2. Previous antibiotic treatment versus no previous antibiotic treatment: any administration of antibiotics 14 days prior to surgery.
3. Early (< 3 months) versus delayed (3–24 months) versus late infection (> 24 months): time between introduction and removal of the implant.

The incidence of microorganisms detected in SFC and PTC was recorded. Coagulase-negative staphylococci (CNS), anaerobes, streptococci and other microorganisms (including fungi among others) were labelled as pathogens of “low virulence”. Methicillin-susceptible and methicillin-resistant *Staphylococcus aureus* (MSSA, MRSA), gram-negative rods and enterococci were labelled as pathogens of “high virulence”. Sensitivity and specificity were calculated using two-by-two contingency tables. McNemar’s test was used to compare proportions among paired samples. Statistical analysis was performed using the SPSS software (IBM Corporation; Armonk, NY, United States).

## Results

A total of 257 cases of potentially infected prostheses were submitted for sonicate fluid analysis between March 2017 and April 2018. Using the EBJIS criteria as the reference standard, 145 and 112 cases were defined as infected and aseptic, respectively. According to the ICM criteria, there were 96 positive, 20 inconclusive and 141 negative cases. Baseline demographics between the two groups are shown in Table 1. The distribution of age, sex and affected joints were similar between cases of PJI and AF. Treatments differed between the two groups. Whereas the two-stage approach (55.2%) was the predominant treatment type in PJIs, the one-stage approach (65.2%) was the most prevalent treatment in AF. In a total of 4 cases (1.6%), salvage procedures were deployed. These included amputation, Girdlestone arthroplasty and local sinus care and suppressive antibiotics.

**Table 1:** Comparison of baseline demographics between PJI and aseptic failure group

		total (*n* = 257)	PJI^1^ (*n* = 145)	AF^2^ (*n* = 112)
age, average			70,0	69,5
sex male		84 (32.7%)	50 (34.5%)	34 (30.4%)
localisation				
	shoulder	21 (8.2%)	10 (6.9%)	11 (9.8%)
	knee	89 (34.6%)	52 (35.9%)	37 (33.0%)
	hip	143 (55.6%)	81 (55.9%)	62 (55.4%)
	other	4 (1.6%)	2 (1.4%)	2 (1.8%)
implant	all	146 (56.8%)	99 (68.3%)	47 (42.0%)
	component	111 (43.2%)	46 (31.7%)	65 (58.0%)
surgery				
	one stage	105 (40.9%)	32 (22.1%)	73 (65.2%)
	two stage	101 (39.3%)	80 (55.2%)	21 (18.7%)
	D&I	47 (18.3%)	30 (20.7%)	17 (15.2%)
	salvage	4 (1.6%)	3 (2.1%)	1 (0.9%)

^1^PJI, periprosthetic joint infection

^2^AF, aseptic failure

Among the 145 cases defined as PJI, 100 were accurately detected by SFC, whereas 45 were missed. Among the 112 cases defined as AF, 101 were accurately detected as negative by SFC, while eleven were falsely positive. This accounted for a sensitivity of 69.0%, and a specificity of 90.2% for SFC. PTC was positive in 91 PJI and accurately negative in 104 AFs, leading to a sensitivity of 62.8% and a specificity of 92.9%. The difference between SFC and PTC sensitivity was statistically significant (*p* = 0.04).

When using the ICM criteria, inconclusive results were excluded from the analysis of diagnostic validity. The sensitivity of SFC and PTC was 87.5% (84/96 cases) and 84.4% (81/96 cases), respectively (*p* = 0.63). Moreover, the specificity was 85.1% (137/161 cases) and 92.5% (149/161), respectively (*p* = 0.05). Among 20 cases with inconclusive findings, six were SFC-positive and four were PTC positive.

In 127 out of 257 cases, both SFC and PTC were negative. In 130 out of 257, a pathogen was detected in either SFC or PTC or both of them. Among these 130 cases, 6 subgroups could be distinguished. The details are depicted in Table 2. The combined sensitivity and specificity of SFC and PTC was 76.6% (111/145) and 83.0% (93/112), respectively.

**Table 2:** results of sonicate fluid culture (SFC) and periprosthetic tissue culture (PTC) in periprosthetic joint infection (PJI) and aseptic failure (AF)

dignity	total	PJI	AF
SFC+^1^ PTC-^2^	35	24	11
SFC+ PTC+,^3^ C^4^	47	47	0
SFC+ PTC+, D,^5^ add SFC^6^	5	5	0
SFC+ PTC+, D, add PTC^7^	12	12	0
SFC+ PTC+, D, diff^8^	12	12	0
SFC-^9^ PTC-	127	34	93
SFC- PTC+	19	11	8
	257	145	112

^1^SFC+, positive result ins sonicate fluid culture

^2^PTC-, negative result in periprosthetic tissue culture

^3^PTC+, positive result in periprosthetic tissue culture

^4^C, concordant

^5^D, discordant

^6^Add SFC, additional pathogen detected by sonicate fluid culture

^7^Add PTC, additional pathogen detected by periprosthetic tissue culture

^8^Diff, different findings

^9^SFC-, negative result in sonicate fluid culture

The subgroup analysis is shown in Table 3. Although sensitivity of SFC was higher than PTC in each subgroup, differences between SFC and PTC were not statistically significant. There was a trend towards higher sensitivity for SFC in late infections (59.5% vs. 45.9%; *p* = .06). Specificity did not differ among subgroups, neither.

**Table 3:** Subgroup analysis and respective diagnostic performance

subgroup		sensitivity (%)			specificity (%)		
		SFC	PTC	*p*^*1*^	SFC	PTC	*p*
overall (EBJIS)		69.0	62.8	*.04*	90.2	92.9	*.65*
overall (ICM 2018)		87.5	84.4	*.63*	85.1	92.5	*.05*
joint							
	*hip (n = 143)*	70.4	63.0	*.24*	91.9	90.3	*1.00*
	*knee (n = 89)*	65.4	53.8	*.15*	91.9	94.6	*1.00*
	*other (n = 25)*	75.0	66,7	*1.00*	76.9	100.0	*/*^*2*^
previous AB^3^ treatment							
	*antibiotics (n = 63)*	72.3	57.4	*.12*	100.0	100.0	*/*
	*no antibiotics (n = 194)*	67.3	61.2	*.36*	88.5	91.7	*.65*
introduction to removal							
	*early (n = 104)*	76.5	67.6	*.36*	94.4	91.7	*1.00*
	*delayed (n = 72)*	65.0	60.0	*.75*	78.1	93.7	*.18*
	*late (n = 81)*	59.5	45.9	*.06*	95.5	93.2	*1.00*

^1^ McNemar’s test of paired proportions. *P* values in italics. A *p* value <.05 indicates statistical significance. SIgnificant values are displayed in fat.

^2^ /, not applicable

^3^ AB, antibiotic

PJA of synovial fluid was performed in 106 PJI and 73 AF. Among PJI, there were 41 positive PJA results (sensitivity: 38.7%). 67 out of 73 PJA were accurately negative (specificity: 91.8%).

**Fig. 2:**
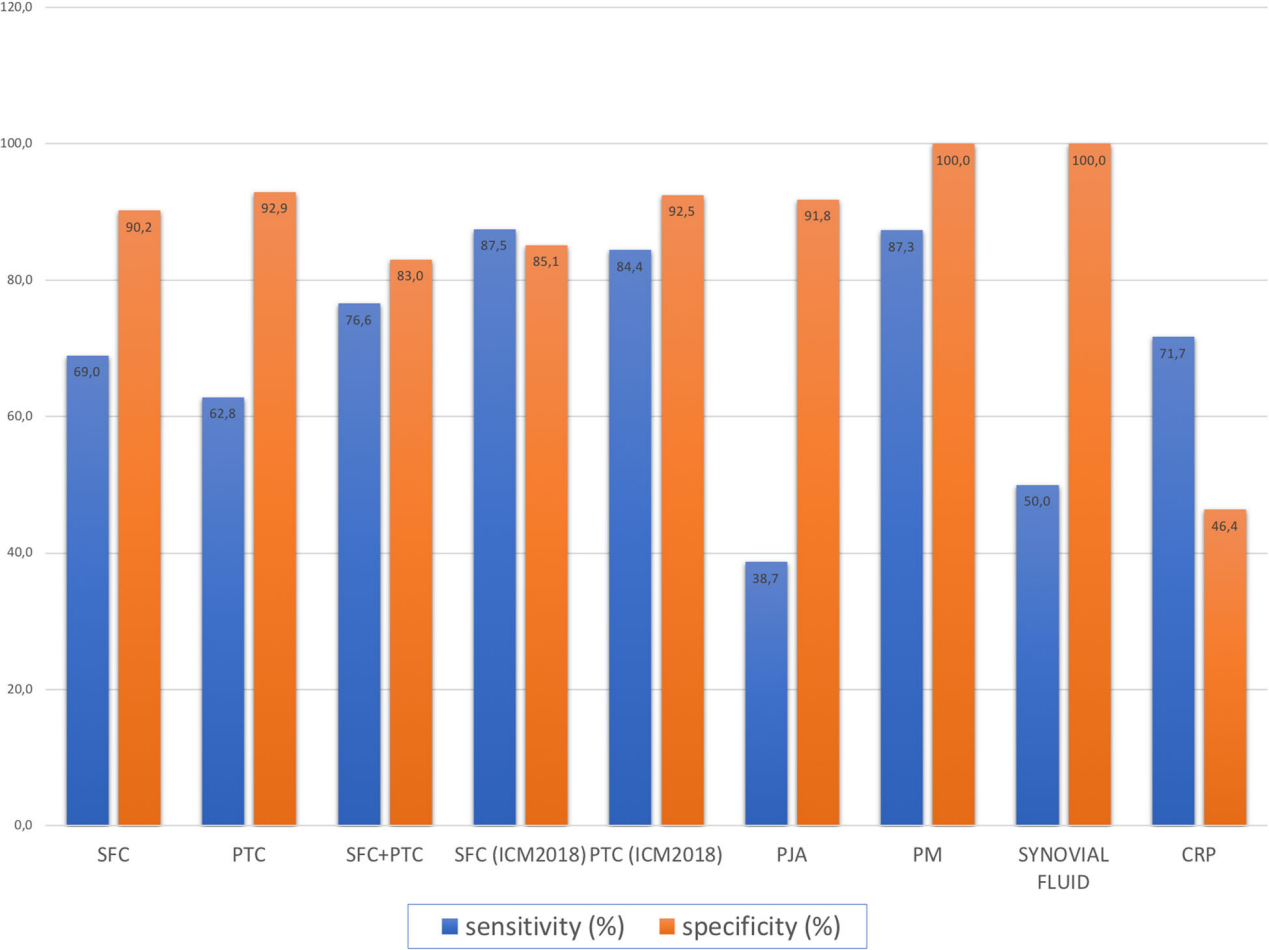
diagnostic performance (sensitivity, specificity) of diagnostic tools deployed Vertical axis represents the value of diagnostic performance in %. On the horizontal axis, the respective tools are depicted. Abbreviations are explained in the text. Blue columns represent sensitivity while orange columns represent specificity

Histopathological analysis of PM was available in 56 PJI and 13 AF. Among the PJI, 48 were indicative of an infection (sensitivity: 85.7%). Samples for WBC and PMN were available in 78 out of 145 PJI and 56 out of 112 AF. Of the 78 PJI, 26 were excluded according to the limitations predetermined by the EBJIS criteria. Of the 52 valid samples, 26 were positive and negative, respectively (sensitivity: 50%). Of the 56 joint aspirations in AF, 24 were excluded. The remaining 32 samples were all negative (specificity: 100%; Fig. 2).

**Fig. 3:**
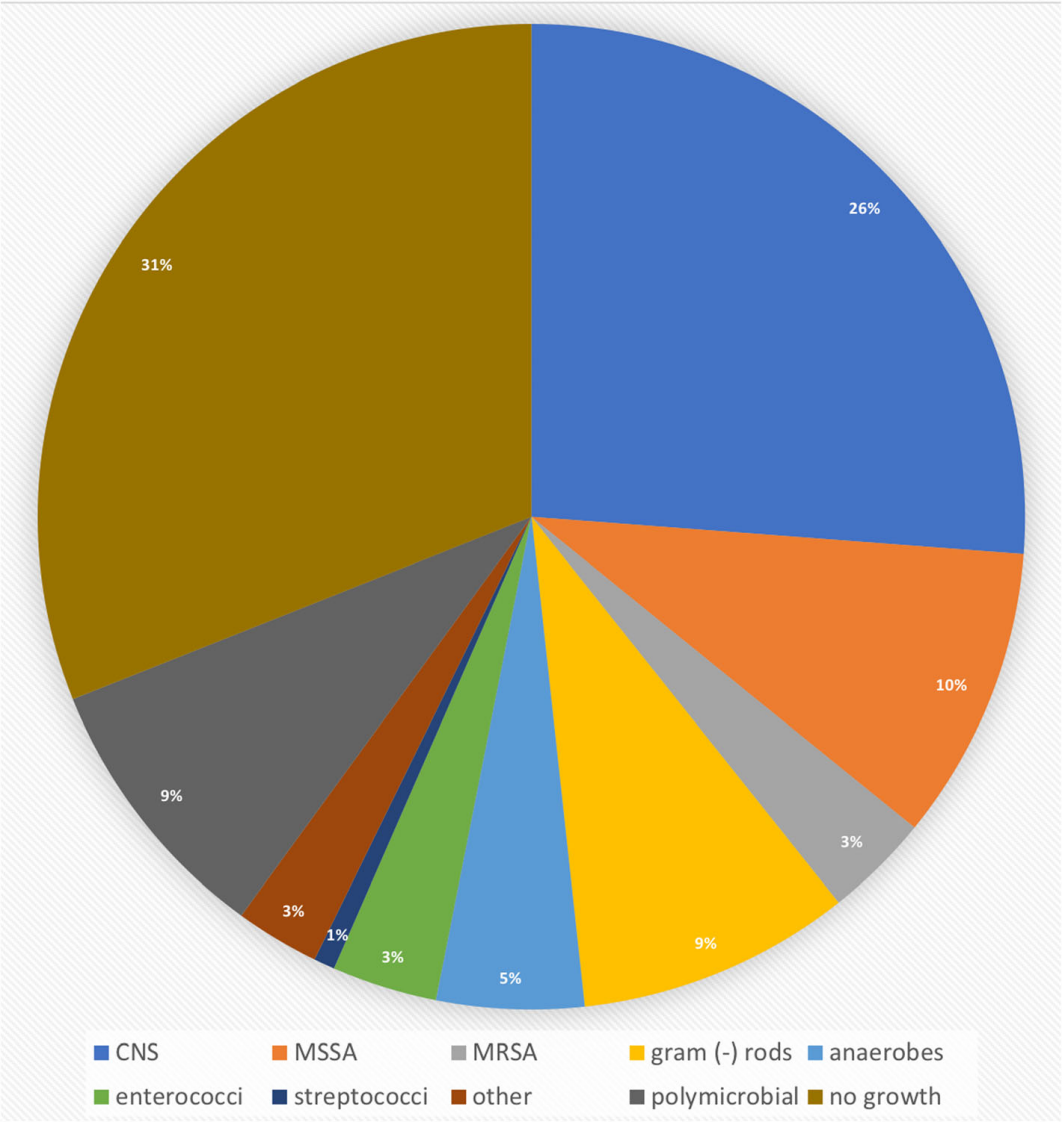
Relative prevalence of pathogens as detected by sonicate fluid culture (SFC) Abbreviations are explained in the text and Table 4. The legend within the figure explains which color represents which entity

The absolute distribution is shown in Table 4 whereas the relative distribution of pathogens detected by SFC is illustrated in Fig. 3. The culture-negative rate was 31.0% (45/145) and 40.0% (58/145) for SFC and PTC, respectively. A polymicrobial result was obtained in 13 (9.0%) and 20 (13.8%) PJI in SFC and PTC, respectively. In early PJI, 48.9% (22/45) and 51.1% (23/45) of pathogens detected through SFC were of high and low virulence, respectively. In delayed and late infections combined, 35.7% (15/42) and 64.3% (27/42) of pathogens detected through SFC were of high and low virulence, respectively. Most notably, 7.8% (6/77) of delayed or late infections included the detection of anaerobes, with *Cutibacterium acnes* as the most common representative (5/6). The corresponding detection rate for PTC was 2.6% (2/77).

**Table 4:** overall distribution of microorganisms and in early, delayed, late infection as detected by sonicate fluid culture (SFC) and periprosthetic tissue culture (PTC)

	early (*n* = 68)		delayed (*n* = 40)		late (*n* = 37)		total (*n* = 145)	
	SFC	PTC	SFC	PTC	SFC	PTC	SFC	PTC
CNS^1^	18	15	14	7	6	8	38	30
*MSSA*^*2*^	*9*	*6*	*1*	*1*	*4*	*5*	*14*	*12*
*MRSA*^*3*^	*2*	*1*	*2*	*2*	*1*	*1*	*5*	*4*
*gram (−) rods*^*4*^	*9*	*6*	*2*	*2*	*2*	*1*	*13*	*9*
anaerobes	1	2	4	2	2	0	7	4
*enterococci*	*2*	*0*	*2*	*1*	*1*	*0*	*5*	*1*
streptococci	1	0	0	2	0	0	1	2
other	3	3	0	1	1	1	4	5
polymicrobial	7	13	1	6	5	1	13	20
no growth	16	22	14	16	15	20	45	58

^1^ *CNS* coagulase-negative staphylococci

^2^ *MSSA* methicillin succeptible *Staphylococcus aureus*

^3^ *MRSA* methicillin resistant staphylococcus aures

^4^ *Gram* (−) rods, gram-negative rods

## Discussion

Diagnosis of PJI is challenging owing to the existing biofilm around implants [12]. Pathogens embedded in a biofilm are enclosed in a polymeric matrix and have altered their phenotype into an extremely resilient form of life, protecting them from antimicrobial and host immune responses [13]. Biofilm bacteria exhibit a markedly higher resistance to antimicrobial killing compared with planktonic bacteria [1].

Sonication was popularized as a diagnostic tool for PJI by Trampuz et al. [14]. During the process, adherent pathogens are dislodged from the surface of the implant through low-frequency ultrasound, preserving their viability [15]. Various studies have shown an improved sensitivity following the use of sonication compared with conventional tissue culture. In a meta-analysis (16 studies) performed by Liu et al. [12], sonication yielded a pooled sensitivity and specificity of 79% (95% confidence interval [CI]: .76–.81) and 95% (95% CI: .94–.96), respectively. The results were similar in a meta-analysis (12 studies) conducted by Zhai et al. [16], showing a pooled sensitivity and specificity of 80% (95% CI: .74–.84) and 95% (95% CI: .90–.98), respectively. Furthermore, a meta-analysis of four studies investigating sonicate fluid in blood culture bottles displayed a sensitivity and specificity of 85% (95% CI: .77–.91) and 86% (95% CI .81 to .91), respectively [17]. However, the reference standards for the diagnosis of PJI differed among the studies included in the meta-analyses.

In our study, SFC showed low sensitivity (69.0%) compared with previous data regarding sonication [12, 16]. Overall, we detected 45 false negative PJI. However, PJI was defined according to the EBJIS criteria, as having a low threshold for the detection of an infection.

When adopting the ICM criteria, the sensitivity and specificity of SFC were comparable with those reported in recent meta-analyses (87.5 and 93.5%, respectively), after the exclusion of inconclusive findings. The discrepancy between the EBJIS criteria and more commonly used criteria (i.e., MSIS) has been reproduced in a recent study performed by Renz et al. [18] which sought to determine the diagnostic value of the alpha defensin lateral flow test using different classification systems. The sensitivity of this test was 84% by MSIS criteria but only 54% by EBJIS criteria.

We found a significantly higher sensitivity of SFC compared with PTC (69.0% vs. 62.8%, respectively; *p* = 0.04). However, the specificity was similar (90.2% vs. 92.9%, respectively; *p* = .65). Our results were in concordance with those of recent studies, that have mostly observed a superior sensitivity of SFC compared with PTC [10, 19, 20]. Two recent studies showed that the sensitivity of SFC was lower than that of PTC [21, 22]. The discrepancy between studies can be attributed to the varying study conditions and definitions of PJI. In particular, the colony-forming unit (CFU) value required to define a positive SFC result differed greatly among studies.

Subgroup analysis showed a superior sensitivity of SFC throughout all subgroups. However, there were no significant differences between SFC and PTC among subgroups. There was a somewhat higher sensitivity for SFC of hip implants when compared with knee implants (hip: 70.4% vs. knee: 65.4%). A possible explanation may be the more frequent use of antibiotic-loaded cement in knee arthroplasty, which may disturb the structure of the biofilm [23]. However, SFC of knee implants might provide a greater advantage against PTC when compared with SFC of hip implants (knee: *p* = .15 vs hip: *p* = .24). Previous administration of antibiotics did not influence the sensitivity of SFC, confirming the findings of comparable studies [24, 25]. However, there was a trend towards higher sensitivity of SFC against PTC (*p* = .12) in the antibiotics groups as opposed to when antibiotics were not administered (*p* = .36). It has to be noted, however, that the antibiotic group was characterized by a relatively small sample size (*n* = 63).

We found both SFC and PTC sensitivity to drop consecutively with delayed and late infections. Low sensitivity in late infections might be a result of EBJIS criteria. A high amount of cases in this group would have been classified as aseptic under alternative definition criteria. Furthermore, we found a strong trend towards higher sensitivity of SFC against PTC in the “late infections” group (59.5% vs. 45.9%; *p* = 0.06) which was in concordance with a recent study by Puig– Verdié et al. [26]. Apparently, in early infection, a majority of microorganisms are expected to not have formed biofilms yet, leading to high detection rates both in SFC and in PTC. In late infection, meanwhile, a biofilm is expected to have formed by most microorganisms and sonication should provide an advantage against conventional culturing.

The prevalence of pathogens was in concordance with current literature. CNS, as in our study (26%), were by far the most common pathogens in various studies across the board [6, 10, 27–29]. The relatively high prevalence of gram-negative rods (9%), especially in early infection (9/81, 11%), is also a finding supported by literature [29]. Moreover, the detection of six anaerobes in 77 cases (7.8%) of delayed and late infections is a finding concordant with literature [28]. Interestingly, out of the six cases, four times anaerobes were detected by SFC alone supporting the notion that these microorganisms are especially susceptible to SFC and may be missed by PTC [15]. With EBJIS criteria, any detection of anaerobes in SFC is a PJI, irrespective of CFU.

We found a comparably high prevalence of culture-negative PJI (31%). In a recent meta-analysis, Reisener et al. [30] reported that the incidence of culture-negative PJI ranged from 7 to 42%. As expected, we found that highly virulent pathogens were more prevalent in early infection than in delayed or late infection, confirming the findings of previous studies [26, 31]. The SFC and PTC results were similar, however, culture-negative results were more frequent in PTC.

The present study has several limitations. Firstly, it is limited by its retrospective nature. Not all elements of the EBJIS PJI criteria were available for each patient. PJA was available in 73 and 65% of PJI and AF cases. WBC/PMN was available in 54 and 50% of PJI and AF cases, respectively. Moreover, PM was available in only 38 and 13% of PJI and AF cases, respectively. An explanation for the lack of data may be the new introduction of standardized principles in our clinic.

Diagnosis according to the ICM criteria may have been biased owing to missing data. The rate of erythrocyte sedimentation among serum markers, as well as leukocyte esterase, alpha defensin and synovial C-reactive protein among synovial markers, were not part of the diagnostic routine. The resulting score contained only points received from available tools.

Besides cases defined as “infected” and “not infected”, the new ICM criteria allow for the detection of “inconclusive” results in patients with a score of 4 or 5. In our study, 20 patients were defined as such. These patients present a real diagnostic challenge and might benefit from molecular diagnostic testing such as next generation sequencing which- of course- was not at our disposal.

Colony counting was not performed with sonication. Especially in SFC(+)PTC(−) cases, measurement of CFU is of high importance in distinguishing between infection and contamination. According to the EBJIS criteria, a CFU count of > 50 CFU/ml is indicative of an infection. In this study, this conflict was solved by interpreting SFC as an additional PTC, and subsequently applying the respective rules. Our method of sonication does not include the step of vortexing. In a subgroup meta-analysis [16], cases that included vortexing within the process of sonication exhibited higher sensitivity (79% vs. 78%, respectively) and higher specificity (96% vs. 85%) versus those that did not.

The strength of this study lies in its relatively high sample size (*n* = 257), which allowed for a reliable comparison between SFC and PTC, and yielded large subgroups for further analyses. Furthermore, definition of PJI and aseptic failure was performed in a very meticulous manner, including all aspects of the respective definition criteria. To our knowledge, this is the first study investigating sonication using the EBJIS criteria as a reference standard for PJI, while simultaneously incorporating the most recent ICM criteria.

## Conclusions

We were able to confirm that SFC has a better sensitivity than PTC based on EBJIS but not on ICM criteria. The specificity, meanwhile, was similar for the two based on both reference standards. According to these findings, improvement of the diagnostic accuracy of sonication is still necessary and the diagnosis of PJI continues to require a combination of diagnostic tools.

## Data Availability

The datasets used and/or analysed during the current study are available from the corresponding author on reasonable request.

## Abbreviations

AF: Aseptic failure(s)
CI: Confidence intervall
CNS: Coagulase-negative staphylococci
CRP: C-reactive protein
EBJIS: European Bone and Joint Society
ESR: Erythrocyte sedimentation rate
ICM: International Consensus Meeting (criteria)
LE: Leucocyte esterase
MRSA: Methicillin-resistant *Staphylococcus aureus*
MSIS: Musculoskeletal Infection Society
MSSA: Methicillin-susceptible *Staphylococcus aureus*
PJA: Preoperative joint aspiration (for synovial fluid culture)
PJI: Periprosthetic joint infection(s)
PM: Periprosthetic membrane
PMN: Polymorphonuclear neutrophil (percentage)
PTC: Periprosthetic tissue culture
SFC: Sonicate fluid culture
SOSSID: Swiss Orthopaedics and the Swiss Society of Infectious Diseases
WBC: White blood cell
